# Postoperative alignment of TKA in patients with severe preoperative varus or valgus deformity: is there a difference between surgical techniques?

**DOI:** 10.1186/s12891-017-1628-8

**Published:** 2017-06-21

**Authors:** Stefan Rahm, Roland S. Camenzind, Andreas Hingsammer, Christopher Lenz, David E. Bauer, Mazda Farshad, Sandro F. Fucentese

**Affiliations:** Orthopaedic Department, Balgrist University Hospital, University of Zurich, Forchstrasse 340, 8008 Zurich, CH Switzerland

**Keywords:** Outliers, Total knee arthroplasty, Severe coronal deformity, Patient specific instrumentation, Computer navigation, Manual instrumentation, Alignment, Measurement

## Abstract

**Background:**

There have been conflicting studies published regarding the ability of various total knee arthroplasty (TKA) techniques to correct preoperative deformity. The purpose of this study was to compare the postoperative radiographic alignment in patients with severe preoperative coronal deformity (≥10° varus/valgus) who underwent three different TKA techniques; manual instrumentation (MAN), computer navigated instrumentation (NAV) and patient specific instrumentation (PSI).

**Methods:**

Patients, who received a TKA with a preoperative coronal deformity of ≥10° with available radiographs were included in this retrospective study. The groups were: MAN; *n* = 54, NAV; *n* = 52 and PSI; *n* = 53. The mechanical axis (varus / valgus) and the posterior tibial slope were measured and analysed using standing long leg- and lateral radiographs.

**Results:**

The overall mean postoperative varus / valgus deformity was 2.8° (range, 0 to 9.9; SD 2.3) and 2.5° (range, 0 to 14.7; SD 2.3), respectively. The overall outliers (>3°) represented 30.2% (48 /159) of cases and were distributed as followed: MAN group: 31.5%, NAV group: 34.6%, PSI group: 24.4%. No significant statistical differences were found between these groups. The distribution of the severe outliers (>5°) was 14.8% in the MAN group, 23% in the NAV group and 5.6% in the PSI group. The PSI group had significantly (*p* = 0.0108) fewer severe outliers compared to the NAV group while all other pairs were not statistically significant.

**Conclusions:**

In severe varus / valgus deformity the three surgical techniques demonstrated similar postoperative radiographic alignment. However, in reducing severe outliers (> 5°) and in achieving the planned posterior tibial slope the PSI technique for TKA may be superior to computer navigation and the conventional technique. Further prospective studies are needed to determine which technique is the best regarding reducing outliers in patients with severe preoperative coronal deformity.

## Background

The generally accepted radiographic goal in total knee arthroplasty (TKA) is the restoration of a neutral mechanical axis (zero degree +/− three degrees). An alignment, which lies beyond this range can lead to premature implant failure [1], abnormal wear [2, 3] and patello-femoral pain [4, 5]. Furthermore, the goal to achieve a neutral mechanical axis has been supported in biomechanical and clinical studies and therefore most authors agree on this concept [1, 5–7].

To date, the three most commonly used techniques for TKA are: 1) the conventional technique with manual instrumentation (MAN) using intramedullary and/or extramedullary jigs to position the cutting blocks; 2) computer navigated instrumentation (NAV) using either an optical or, recently introduced, an electromagnetic wireless system to intraoperatively position the cutting jigs correctly and 3) patient specific instrumentation technique (PSI) using individualized cutting jigs designed from 3D images from the patient’s anatomy (based on a preoperative computer tomography (CT) scan or magnetic resonance imaging (MRI)).

The conventional technique has shown that outliers (>3°) may be produced at a rate of up to 32% [8, 9], encouraging more predictable techniques to be developed. The introduction of computer navigation has been associated with fewer outliers [8], but on the other hand, there is conflicting data regarding the radiographic accuracy in the coronal alignment. Some studies have shown that computer navigation provides no significant decrease in outlier incidence [10, 11] and in some cases, may even increase the proportion of outliers [12–14]. Overall, the majority of orthopaedic surgeons have not been convinced of computer navigation due to the uncertainty of a true benefit and potential downsides such as longer operating times, issues with bicortical tibial and femoral pins (iatrogenic fractures, infection) and higher costs [15–17].

An increasingly popular development in TKA is patient specific instrumentation (PSI), based on preoperative CT or MRI. Potential benefits include increased efficiency and accuracy with no additional intramedullary canal violation and less blood loss. However, there are also conflicting results concerning radiographic accuracy compared to the two other techniques [18–24].

It is well known that the severity of the preoperative coronal alignment is associated with a worse postoperative result independent of the surgical technique [25, 26]. There are no studies published, which analyse the postoperative radiographic accuracy regarding the three previously mentioned instrumentation techniques with respect to severe preoperative coronal deformity. Therefore, the rational of the present study is to retrospectively analyse the postoperative radiographic alignment of the three different techniques in patients with severe coronal deformity (≥ 10° varus or valgus).

The hypothesis was that computer navigation or PSI technique would provide better radiological accuracy than the conventional technique.

### Methods

The study was approved by the Cantonal Ethics Committee of Canton Zurich (KEK-Zurich) (Institutional review board (IRB) No. 2015–0560). This was a retrospective study with retrospective data collection. We identified patients who had undergone primary TKA for osteoarthritis in the time period from 2004 until 2012. Overall 1063 primary TKAs were identified including 269 SAL UC; Self Aligning Ultra Congruent (Zimmer Inc., Warsaw, IN, USA) and 466 NexGen Legacy Posterior Stabilized Flex (Zimmer Inc., Warsaw, IN, USA) and 328 GMK; Global Medacta Knee (Medacta International S.A., Castel San Pietro, Switzerland).

The charts and radiographs of these patients were analysed and grouped into either manual instrumentation (MAN) using an intramedullary rod for the femoral and an extramedullary guide for the tibial cut, computer-navigation (NAV) (Navitrack surgical navigation system, Zimmer, Warsaw, IN, USA) or CT based patient specific instrumentation (MyKnee, Medacta International S.A., Castel San Pietro, Switzerland) [19]. Patients in the PSI group underwent preoperative CT scan including the hip and the ankle. The goal in all cases was to achieve a neutral coronal alignment.

Inclusion criteria included a complete pre- and postoperative radiograph set with a preoperative varus or valgus of 10 ° or more. Primary TKA using a semi-constrained or constrained type of TKA were excluded.

The three groups were analysed and compared to each other. Furthermore, a subgroup analysis regarding surgeon’s experience and implant used was performed. In Table 1 the distribution of each group regarding the number of patients, surgeon experience, implant and varus/ valgus deformity is shown.

**Table 1:** The preoperative characteristics are depicted in this table. The *p*-value shows the homogeneity of the three groups regarding age, BMI, gender and preoperative coronal deformity and significant difference regarding surgeons experience and used implant

	All	Group MAN	Group NAV	Group PSI	*p* = *
n=	159	54	52	53	n.a.
age (years mean (SD))	70 (10.1)	71 (9.1)	69 (10.)5	69 (10.6)	0.56
BMI (kg/m^2^; mean (SD))	30 (5.8)	30 (5.8)	31 (5.3)	29 (6.2)	0.482
Gender (n = f/m)	98/61	34/20	33/19	31/22	0.632
Varus n=	118	36	41	41	0.292
Varus degrees mean (SD)	13.0 (2.8)	12.6 (2.1)	13.0 (3.0)	13.8 (2.9)	0.13
Valgus n=	41	18	11	12	0.292
Valgus degrees mean (SD)	−13.0 (2.9)	−13.3 (3.6)	−12.8 (2.2)	−11.3 (1.7)	0.13
Surgeon’s experience >100 TKA	104	30	24	50	<0.001
Surgeon’s experience 50 to 100 TKA	55	24	28	3	<0.001
Zimmer NexGen n=	88	39	49	0	<0.001
Zimmer SAL n=	15	15	0	0	< 0.001
Global Medacta Knee n=	56	0	3	53	<0.001

*SD* standard deviation

∗ The Kruskal-Wallis test was applied to compare the distribution of continuous variables among groups and the Chi-square test was employed to compare the distribution of nominal variables among groups

### Radiographs

The preoperative radiographic assessment consisted of X-rays of the knee (ap / lateral / patella axial view) and a standing long-leg radiograph for assessment of the correct coronal alignment. For the PSI group an additional CT scan was performed according to a special protocol, which included the hip and ankle. Between 6 weeks and 3 months postoperatively, standard radiographs with long-standing X-ray were routinely performed in our outpatient clinic.

In 6 patients there was no standing long-leg radiograph available between 6 weeks and 3 months. Therefore in these 6 patients the postoperative standing long-leg radiograph were performed between 6 and 18 months postoperatively.

All measurements were performed by two senior orthopaedic residents (A.H. and C.L.) and the inter-rater reliability was calculated. The mechanical axis of the lower extremity in the frontal plane was measured in the pre- and postoperative standing long-leg radiographs. The measurement was from the centre of femoral head to the intercondylar notch of the distal femur, and the centre of the proximal tibia to the centre of the ankle.

The posterior tibial slope was measured in the lateral postoperative X-rays according to Faschingbauer et al. [27].

### Outliers

A neutral postoperative mechanical alignment was defined within ±3°. An outlier was defined as a mechanical axis with more than 3° varus or valgus. The target posterior tibial slope varied between manufacturer designs. Therefore an outlier was defined as 3° or more than the targeted posterior tibial slope.

### Statistics

Statistical analyses were performed with PRISM 5 Graphpad for MAC OS. Normally distributed variables are reported as mean with standard deviations (SD). The distribution of outlier between groups was analysed using fishers exact test. The unpaired and paired students-t-test as well as the Chi-square test, ANOVA (analysis of variance) or ANOVA by ranks as appropriate for comparison between more than two groups were employed for intergroup and Intra-group comparison, respectively. The interrater reliability of continuous variables (varus/valgus/slope) was determined using interclass correlations (ICC) derived from 2-way mixed-effects ANOVA (average measures). Ninety-five percent confidence intervals (CI) are reported for ICC. The criteria of Landis and Koch were used for the magnitude of the reliability coefficient: 0 to 0.2 = poor, 0.21 to 0.4 = fair, 0.41 to 0.6 = moderate, 0.61 to 0.8 = substantial, 0.81 to 1.0 = excellent agreement. Statistical level of significance was defined with a *p* < 0.05.

## Results

Pre- and postoperative measurement of varus and valgus and sagittal alignment had excellent agreement between both readers (varus: ICC: .995, CI: .993–.996, valgus: ICC: .988, CI: .983–991, sagittal alignment: ICC: 0.955 CI: 0.922–0.974). We therefore decided to use the mean of the two readers (A.H. and C.L.).

### Coronal alignment

**Fig. 1:**
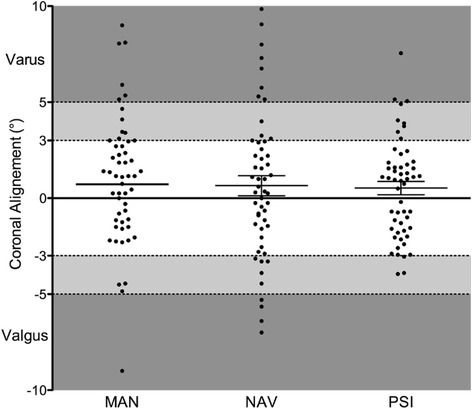
Here are all patients’ coronal alignment depicted with a dot and the distribution is visualized. The PSI group has the smallest variability

**Fig. 2:**
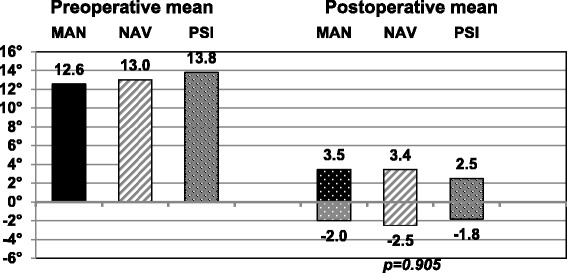
The mean postoperative results of all patients with a preoperative varus deformity are depicted. There were no significant differences shown between the three groups.

**Fig. 3:**
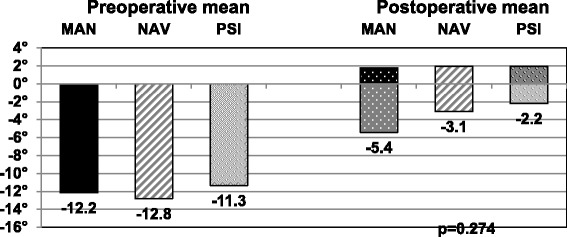
The mean postoperative results of all patients with a preoperative valgus deformity are depicted. Again, there were no significant differences seen between the three groups

The overall mean postoperative varus / valgus deformity was 2.8° (range, 0 to 9.9°; SD 2.3) / 2.5° (range, 0 to 14.7°; SD 2.3), respectively. The postoperative results are summarized completely in Fig. 1 and show the larger variability in the MAN and the NAV group compared to the PSI group. In Fig. 2 the pre- to postoperative results are depicted of the patients with a preoperative varus deformity and in Fig. 3 with a preoperative valgus deformity, respectively. There are no significant differences found between the groups pre- or postoperatively. Further, the pre-to postoperative correlation did not reach significance in any of the three groups. Group MAN *r* = 0.219, group NAV *r* = 0.102, group PSI *r* = 0.104. This means that a preoperative varus or valgus deformity did not influence the postoperative result regarding varus or valgus deformity significantly.

**Fig. 4:**
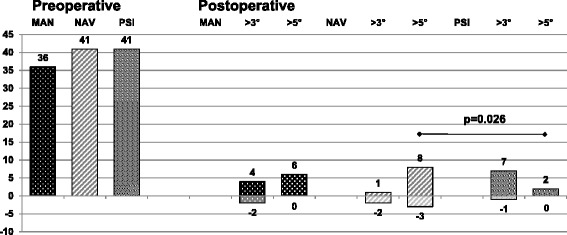
The results summarized of all the patients with a preoperative varus deformity showing postoperative outliers (>3°) and severe outliers (>5°). The PSI group shows significantly less outliers than the NAV group

**Fig. 5:**
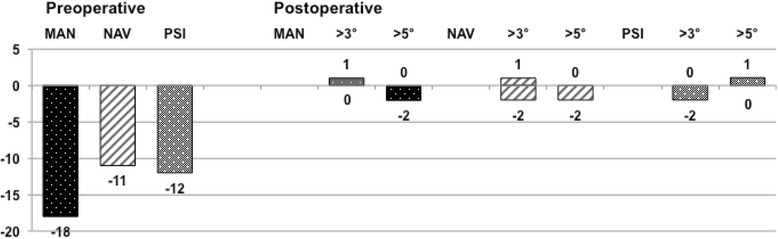
The results summarized, similar to Fig. 4, all the patients with a preoperative valgus deformity showing postoperative outliers and severe outliers. No significant differences were detected

The overall coronal outliers defined as >3° reached 30.2% (48 /159). Table 2 shows the main results with the statistical significance. The PSI group showed the fewest number of severe outliers (>5°) compared to the other groups and showed statistical significance between PSI and NAV group; *p* = 0.011. The PSI group, including only the patients with preoperative varus deformity again showed fewer severe outliers >5° compared to the NAV group; *p* = 0.026. The complete results regarding the separate preoperative varus and valgus groups are depicted in Figs. 4 and 5.

**Table 2:**
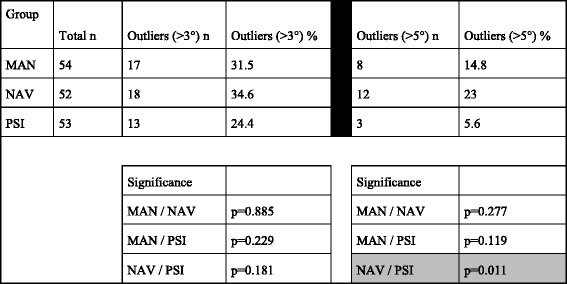
Overall results showing the postoperative outliers in the coronal plane

Statistical significance was calculated by Fisher Exact Test. Gray shaded = significant

### Subgroup analysis

The three groups were analysed separately after excluding the patients who had been operated by an attending with 50 to 100 TKA ending up with three homogenous groups regarding the surgeon’s experience. There were no statistical differences found in the postoperative coronal outcome between the three subgroups and therefore no statistical differences compared to the complete group.

The three groups were analysed separately after excluding the different types of TKA ending up with three homogenous groups regarding the TKA type. These results showed no significant differences compared to the result of the three complete groups.

### Sagittal alignment

The outliers defined as >3° off the planned posterior tibial slope are shown in Table 3. Two pairs show statistical significance; MAN / NAV: *p* = 0.007, MAN / PSI: *p* = 0.006.

**Table 3:**
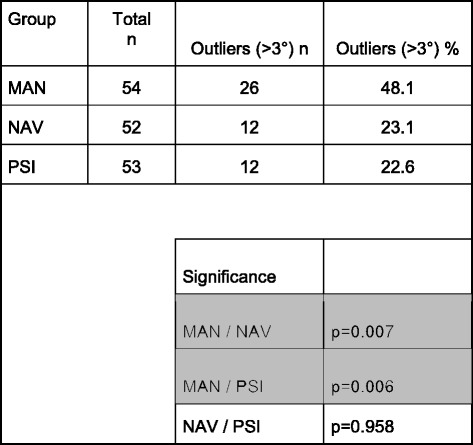
Overall results showing the postoperative outliers in the sagittal plane regarding the posterior tibial slope. An outlier was defined when more than 3° difference to the planned posterior tibial slope

Statistical significance was calculated by Fisher Exact Test. Gray shaded = significant

## Discussion

The most significant finding of the present study is that the overall outlier rate is rather high at 30.2% (48 /159), in patients with a severe preoperative varus or valgus deformity of 10° or more regardless of the surgical technique. Between the three groups there was no significant difference regarding postoperative coronal accuracy. Therefore our hypothesis has to be rejected. However, in this study the preoperative coronal deformity did not correlate with the postoperative result regarding outliers or radiographic accuracy in varus or valgus. This can be well explained since the patients with less than 10° coronal deformities were excluded. Several authors have identified that a severe, preoperative deformity is associated with a relatively poor postoperative alignment and therefore also a worse clinical long-term outcome [25, 26, 28, 29]. Comparing our results to the literature, the overall outlier range is higher in the patient population with a severe preoperative coronal deformity. The overall outliers (>3°) reached 30.2% (48 /159), which is over the value of most of the studies found in literature regardless of the surgical technique. This is a very important finding that a higher outlier rate occurs in severe deformity and this information maybe should be shared with our patients.

The PSI group had the fewest proportion of outliers (>3° for the frontal mechanical axis) with 24.4% compared to 31.5% in the MAN group and 34.6% in the NAV group. Although not statistically significant, there was a trend towards a benefit in the PSI group. When using a threshold value of >5°, defined to be severe outliers, the results showed larger differences reaching statistical significance with a *p* = 0.011 between the PSI and the NAV group. Further, the accuracy reaching the planned posterior tibial slope was statistically worse in the MAN group with 48.1% outliers (>3° of the planned value) compared to the PSI group with 22.6% (*p* = 0.006) and to the NAV group with 23.1% (*p* = 0.007).

The PSI group has significantly fewer severe outliers due to the precise preoperative planning and accurate cutting guides which should result in a 0° mechanical axis. In our hands this technique seems to be the most accurate in this patient group. On the other hand a preoperative CT scan is necessary in all the patients who received a PSI TKA and therefore this is a potential appreciable downside. However, the outlier range of 24.4% is still too high when comparing the results with a study performed in our hospital with the same TKA and PSI technique. In this group with less severe coronal deformity the outlier group represented 12% [19]. Two other studies using the same PSI system showed outliers of 10% [18] and 37% [30]. The relatively high outlier proportion in the study of Ensini et al. [30] may be explained by a small study population and the associated issues with learning a new PSI technique. Therefore, this huge variability of the different studies is explained. The accuracy regarding the posterior tibial slope is similar in the PSI and the NAV group but interestingly here the MAN group was significantly worse than the other two.

Our findings suggest that the computer navigation did not improve accuracy in this study population. This is in contrast to other authors reporting fewer outliers with computer navigation [8]. The reasons are multifactorial and not fully understood. One potential reason for this discrepancy may be due to the varied experience between the surgeons. Less experienced surgeons who performed fewer than 100 represented 28 of the 52 cases with computerized navigation.

In a study performed by Carli et al. [31] comparing two different computer navigation systems, the results showed a significant difference. However, in both study groups there were rather high numbers of outliers, 24 and 32%, respectively.

In the study of Willcox et al. [32] the intraoperative alignment according to the navigation differed from the alignment measured using a standing long-leg radiograph.

There are considerable limitations to this study. First, the standing long-leg radiograph can mimic a false coronal deformity if the patient does not fully extend their knee and/or a rotational malalignment is present [33, 34]. We do not believe that it is justified to perform a postoperative CT scan in order to address this concern. Secondly the groups are not in all aspects homogenous. However, the subgroup analysis did not change the main conclusion and we therefore believe that this limitation is negligible.

### Conclusion

In severe varus / valgus deformity the three surgical techniques demonstrated similar postoperative radiographic alignment. However, in reducing severe outliers (> 5°) and in achieving the planned posterior tibial slope the PSI technique for TKA may be superior to computer navigation and the conventional technique. Further prospective studies are needed to determine which technique is the best regarding reducing outliers in patients with severe preoperative coronal deformity.

## Abbreviations

CI: Confidence intervals
CT: Computer tomography
GMK: Global medacta knee
ICC: Interclass correlations
IRB: Institutional review board
MAN: Manual instrumentation
MRI: Magnetic resonance imaging
NAV: Computer navigated instrumentation
PSI: Patient specific instrumentation
SAL UC: Self aligning ultra congruent
TKA: Total knee arthroplasty
